# RETAIN: A Board Game That Improves Neonatal Resuscitation Knowledge Retention

**DOI:** 10.3389/fped.2019.00013

**Published:** 2019-01-31

**Authors:** Maria Cutumisu, Siddhi D. Patel, Matthew R. G. Brown, Caroline Fray, Patrick von Hauff, Thomas Jeffery, Georg M. Schmölzer

**Affiliations:** ^1^Centre for the Studies of Asphyxia and Resuscitation, Neonatal Research Unit, Alberta Health Services, Royal Alexandra Hospital, Edmonton, AB, Canada; ^2^Centre for Research in Applied Measurement and Evaluation, University of Alberta, Edmonton, AB, Canada; ^3^Department of Computing Science, University of Alberta, Edmonton, AB, Canada; ^4^Faculty of Science, University of Alberta, Edmonton, AB, Canada; ^5^Faculty of Medicine and Dentistry, Academic Technology, University of Alberta, Edmonton, AB, Canada; ^6^Department of Pediatrics, Faculty of Medicine and Dentistry, University of Alberta, Edmonton, AB, Canada

**Keywords:** infant, newborn, delivery room, neonatal resuscitation, neonatal simulation, board game

## Abstract

**Background:** The current resuscitation guidelines recommend frequent simulation based medical education (SBME). However, the current SBME approach is expensive, time-intensive, and requires a specialized lab and trained instructors. Hence, it is not offered routinely at all hospitals. We designed the board game “RETAIN” to train healthcare providers (HCPs) in neonatal resuscitation in a cost-friendly and accessible way.

**Objectives:** To examine if a board game-based training simulator improves knowledge retention in HCPs.

**Methods:** “RETAIN” consists of a board using an image of a baby, visual objects, adjustable timer, monitors, and action cards. Neonatal HCPs at the Royal Alexandra Hospital were invited to participate. Participants completed a written pre-test (resuscitation of a 24-week infant), then played the board game (starting with a tutorial followed by free playing of three evidence-based neonatal resuscitation scenarios). Afterwards, a post-test with the same resuscitation scenario and an opinion survey was completed. The answers from the pre- and post-test were compared to assess HCPs' knowledge retention.

**Results:** Thirty HCPs (four doctors, 12 nurses, and 14 respiratory therapist) participated in the study. Overall, we observed a 10% increase in knowledge retention between the pre- and post-test (49–59%, respectively). Temperature management showed the most knowledge gain between the pre- and post-test (14–46%, respectively). Placement of a hat (10–43%), plastic wrap (27–67%), and temperature probe (7–30%) improved between the pre- and post-test.

**Conclusion:** Knowledge retention increased by 12% between pre- and post-test (49–61%, respectively). The improvement in performance and knowledge supports the use of board game simulations for clinical training.

## Introduction

The majority of newborns make the transition from fetal to neonatal life without help. However, about 10% of newborn infants require interventions (1). The most commonly applied interventions include stimulation, suction, drying, and respiratory support (2–4). The delivery room is a stressful environment where decisions are made quickly and resuscitators must possess outstanding cognitive, psychomotor, and communication skills to identify problems, analyze complex scenarios, generate solutions, and integrate a large amount of data into useful information under elevated time pressure (5). This situation leads often to human errors and deviations from proper protocol (2–4). Analyses of the main reasons for the occurrence of human error in neonatal resuscitation point to a lack of practical learning experiences (6, 7). This is highlighted by the neonatal training paradox of high-acuity, low-occurrence (HALO) situations (8). To address this issue, the recent neonatal resuscitation guidelines recommend simulation-based medical education (SBME) (9). Studies have reported that such SBME activities can enhance knowledge retention and decrease human errors in real-life clinical situations (9).

However, the current SBME approach is expensive, time-intensive, and requires a specialized lab and trained instructors (10, 11). Hence, it is not offered frequently enough for development of competency and for supporting knowledge retention. Moreover, while SBME has been shown to improve performance initially after training (12, 13), both cognitive and technical skills significantly deteriorate within months (11). Therefore, other methods of training to improve knowledge retention and decision-making are needed. We designed the educational game platform “RETAIN” to train healthcare providers (HCPs) in neonatal resuscitation in a cost-friendly and accessible way. RETAIN consists of a computer role-playing game (10, 14) and a board game, as tools that complement the physical SBME to improve knowledge retention during neonatal resuscitation in the delivery room. We recently reported that growth mindset moderated the relation between HCPs' performance in the RETAIN video game simulator and the time since their last refresher Neonatal Resuscitation Program (NRP) course (14). The aim of the current study was to examine if the RETAIN board game could improve knowledge retention during simulated neonatal resuscitation. We hypothesized that HCPs would achieve higher post-test scores, hence knowledge retention, compared to the pre-test.

## Methods

The study was performed at the simulation lab at the Center for the Studies of Asphyxia and Resuscitation, Edmonton, Canada. The Center for the Studies of Asphyxia and Resuscitation is integrated within the Neonatal Intensive Care Unit (NICU) at the Royal Alexandra Hospital, Edmonton, a tertiary perinatal center admitting more than 350 infants with a birth weight of <1,500 g to the neonatal nursery annually. HCPs trained in NRP, including registered nurses, respiratory therapists, neonatal nurse practitioners, neonatal consultants, and neonatal fellows, were recruited from the Royal Alexandra Hospital. The study was approved by the Human Research Ethics Board at the University of Alberta (Pro00075351), and written informed consent was obtained from the HCPs participants prior to participation.

## The RETAIN Board Game

**Figure 1:**
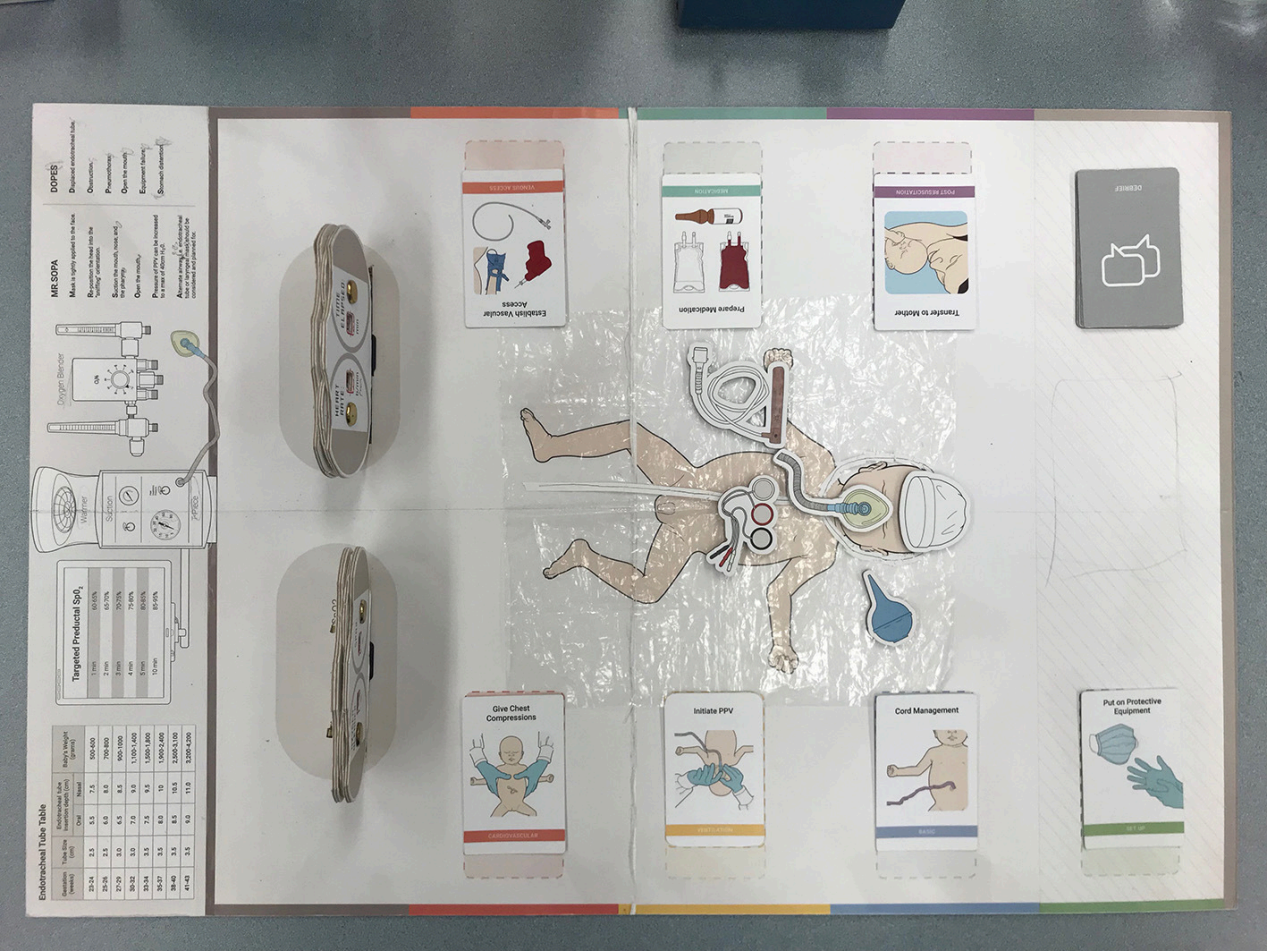
The “RETAIN” board game with action cards, visual objects, reference tables, mnemonics, and adjustable timers and monitors.

**Figure 2:**
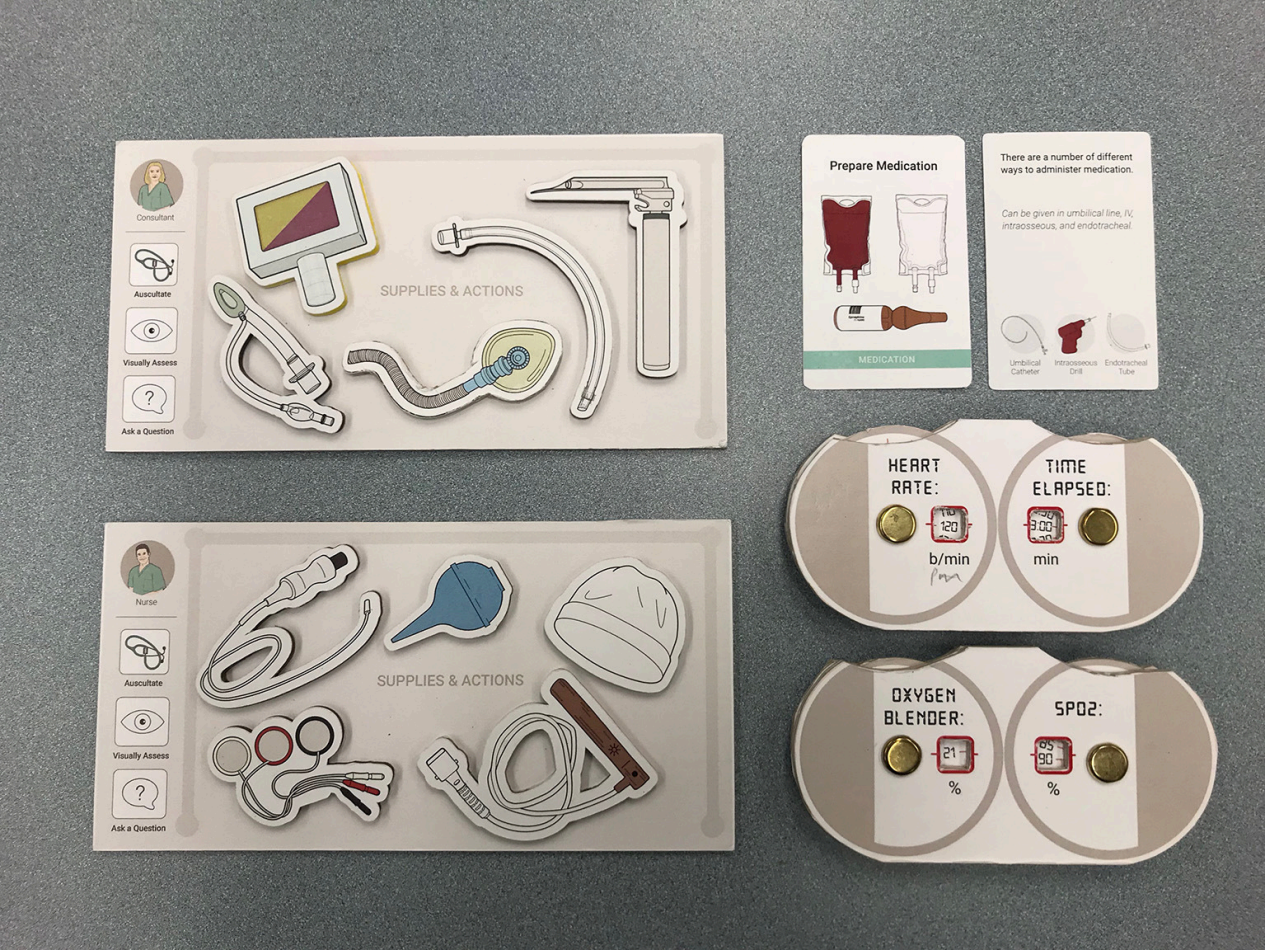
Excerpt of a personal board indicating the player's role and visual objects, action cards, and adjustable timers and monitors within the board game RETAIN.

The *RETAIN board game* (https://www.playretain.com, RETAIN Labs Medical Inc. Edmonton, Canada) is a table-top simulation serious board game where HCPs are presented with a series of evidence-based scenarios based on real-life delivery room resuscitations from the Royal Alexandra Hospital, Edmonton, Canada. During the game, HCPs must prepare and perform resuscitations using equipment, supplies, action and debrief cards; along with adjustable monitors (Figure 1). The game can be played individually or with up to 4 players as an interdisciplinary team (e.g., doctors, trainees, nurse, or respiratory therapist). The game also includes action and debriefing cards, and visual objects representing tools, equipment and medication, which are placed on the board. Each action card has an image representing an action (e.g., prepare medication, give chest compressions etc.) along with text representing each action on the front side (Figure 2).

The action cards included (i) *Set-up* cards, which function as checklists prior to delivery; (ii) *Assign Roles* cards to assign each player's role; (iii) *Basic* cards (e.g., dry, stimulate, assess breathing); (iv) *Ventilation* cards (e.g., Provide Continuous Positive Airway Pressure); (v) *Cardiovascular* cards (e.g., Give Chest Compressions); (vi) *Venous Access* cards (e.g., Establish Vascular Access); (vii) *Medication* cards (e.g., Administer Medication); and (viii) *Post Resuscitation* cards (e.g., Admit to NICU, or Transfer to Mother) (Figures 1, 2). Helpful information about the action (e.g., how to perform the action or helpful visual cues) is presented on the reverse side (Figure 2). Furthermore, action cards are associated with one or more visual objects (Figures 1, 2). At the start of the game, players receive a personal board with their role indicated on the corner, and space to keep the visual objects and action cards assigned to their role (Figure 2). The board game also has reference tables (e.g., oxygen saturation targets) and mnemonics (e.g., MR. SOPA [M (mask adjustment), R (reposition airway), S (suction mouth and nose), O (open mouth), P (pressure increase), A (alternate airway] and DOPES [Displacement of the endotracheal tube (ETT), Obstruction of the ETT, Pneumothorax, Equipment failure, Stomach distension]) at the top of the game board (Figure 1). There are adjustable timers and monitors (e.g., Apgar clock, Heart rate), which are adjusted as each scenario proceeds (Figures 1, 2).

## Study Design

Each participant was asked to complete a demographic information questionnaire (e.g., last NRP-course, years of experience, etc.) and a pre-test to assess each HCP's current knowledge. The pre-test (Appendix 1) consisted of an evidence-based neonatal resuscitation scenario of a 24-week premature infant, with open-ended questions where HCPs had to explain which steps they would take to stabilize the infant. Each participant then played the “RETAIN” board game simulator, which started with a tutorial (a stepwise approach to review the current neonatal resuscitation guidelines) to review the current neonatal resuscitation algorithm moderated by a facilitator (SDP). After the tutorial, each HCP was given the opportunity to play three evidence-based resuscitation scenarios. Afterwards, a post-test (Appendix 1) using the same scenario as the pre-test was used to assess knowledge retention. In addition, each participant completed a post-simulation questionnaire to assess the player's mindset (e.g., How much do you agree with the following statements? You can always change how good you are at your job or You can get better at your job with practice) using a Likert scale (1 = Strongly Disagree to 5 = Strongly agree).

At the start of the gameplay, the participant acted as the team leader and could choose any role (e.g., consultant, nurse, respiratory therapist, nurse practitioner, trainee, or midwife). There were several standardized players (Ramin Lagarde, Georg Schwarzl, Deandra Luong, and Sehar Rajani), who took on support roles (e.g., nurse, respiratory therapist, trainee, or midwife) during the game, which allowed to play the game as a team.

At the start of the game, the facilitator (who was a researcher, not a participant) informed the participant about an imminent delivery. A brief history including maternal and pregnancy course was verbally given to allow for delivery preparation. Throughout the game, the participant acted as team leader and verbally instructed the standardized players to place the necessary equipment onto the board, as well as play the required action cards. Throughout the game, players use their action cards in response to the current game situation in the scenario, while the facilitator adjusts values on the monitors and timers accordingly. The adjustments by the facilitator were directed by the scenario information in the instruction booklet.

## Statistical Analyses

### Predictor Variable

The Pre-test represents the participant's cumulative score across all actions, interventions, or tasks described by the participant for the written test. Outcome Variable: The Post-test represents the participant's cumulative score across all actions, interventions, or tasks described by the participant for the board game. The maximum score for each participant, when answering all actions, interventions and tasks correctly, was 16 points, with a range from 0 to 16. Participants were assigned one point for each correct actions, interventions, or tasks on the pre- and post-test.

We also used the individual measures corresponding to each of the actions, interventions, or tasks described by the participant of the pre-test and the corresponding actions, interventions or tasks of the post-test.

The data are presented as mean (SD) for normally distributed continuous variables and median (interquartile range—IQR) when the distribution was skewed. For all respiratory parameters, continuous values during CPR were analyzed. The data was tested for normality and compared using a repeated-measures analysis of variance (ANOVA) with a Greenhouse-Geisser correction to compare the overall performance of the participants between pre- to post-test, Student's *t-*test for parametric and Mann-Whitney *U*-test for nonparametric comparisons of continuous variables, and χ^2^ for categorical variables. *P*-values are 2-sided and *p* < 0.05 was considered statistically significant. Statistical analyses were performed with SigmaPlot (Systat Software Inc., San Jose, USA).

## Results

We recruited 30 HCPs (24 females and 6 males; four doctors, 12 nurses, and 14 respiratory therapists) who were all NRP-trained and had completed an NRP refresher course within the last 24 months of the current study.

### Overall Performance

Overall, we observed a 12% increase in performance between the pre- and post-test (49–61%, respectively). The participants' mean scores significantly increased [*F*_(1, 29)_ = 21.41, *p* < 0.001, $\eta_{\text{p}}^{2}$ = 0.42] from the pre-test (*M* = 7.87, *SD* = 2.18, *SE* = 0.40, 95%[7.05, 8.68]) to the post-test (*M* = 9.77, *SD* = 2.67, *SE* = 0.49, 95%[8.77, 10.76]). Therefore, we can conclude that the board game improved participants' performance on the given neonatal resuscitation scenario from pre-test to post-test.

### Temperature Management

Temperature management showed the most knowledge gain between the pre- and post-test (14–46%, respectively). Placement of a hat (10–43%), plastic wrap (27–67%), and temperature probe (7–30%) improved between the pre- and post-test. A more detailed repeated-measures ANOVA revealed that temperature management showed a significant increase between pre- and post-test. Specifically, placement of a hat increased significantly: *F*_(1, 29)_ = 11.15, *p* < 0.01, $\eta_{\text{p}}^{2}$ = 0.28, 95%CI [−0.54, −0.13] from pre-test (*M* = 0.10, *SD* = 0.30) to post-test (*M* = 0.43, *SD* = 0.50). Similarly, using plastic wrapping to maintain temperature significantly increased: (*F*_(1, 29)_ = 19.33, *p* < 0.001, $\eta_{\text{p}}^{2}$ = 0.40, 95%CI[−0.59, −0.21]) from pre-test (*M* = 0.27, *SD* = 0.45) to post-test (*M* = 0.67, *SD* = 0.48). Finally, attaching a temperature probe to continuously monitor the baby's temperature significantly increased: (*F*_(1, 29)_ = 8.83, *p* < 0.01, $\eta_{\text{p}}^{2}$ = 0.23, 95%CI[−0.39, −0.07]) from the pre-test (*M* = 0.07, *SD* = 0.25) to the post-test (*M* = 0.30, *SD* = 0.47).

### Monitoring

Attaching pulse oximeter probe (SpO_2_) (57–77%) and attaching electrocardiographic (ECG) leads (47–73%) improved from the pre-test to the post-test. HCPs significantly improved on attaching both the SpO_2_ and ECG leads when pre- and post-test were compared. SpO_2_ (*F*_(1, 29)_ = 5.12, *p* = 0.03, $\eta_{\text{p}}^{2}$ = 0.15, 95%CI[−0.38, −0.02]) from pre-test (*M* = 0.57, *SD* = 0.50) to post-test (*M* = 0.77, *SD* = 0.43). ECG: (*F*_(1, 29)_ = 6.27, *p* = 0.02, $\eta_{\text{p}}^{2}$ = 0.18, 95%CI[−0.48, −0.05]) from pre-test (*M* = 0.47, *SD* = 0.51) to post-test (*M* = 0.73, *SD* = 0.45).

### Respiratory Management

There was a negative effect on the task of assessing breathing between the pre- and post-game test (*F*_(1, 29)_ = 5.12, *p* = 0.03, $\eta_{\text{p}}^{2}$ = 0.15, 95%CI[0.02, 0.38]) from pre-test (*M* = 0.70, *SD* = 0.47) to post-test (*M* = 0.50, *SD* = 0.51). Analyses also revealed that HCPs significantly improved at correctly deciding when to provide continuous positive airway pressure: (*F*_(1, 29)_ = 5.80, *p* = 0.02, $\eta_{\text{p}}^{2}$ = 0.17, 95%CI[−0.31, −0.02]) from pre-test (*M* = 0.80, *SD* = 0.41) to post-test (*M* = 0.97, *SD* = 0.18).

### Admission to NICU

Admission to NICU increased from 0–47% from the pre-test to the post-test. Finally, we observed a significant increase on admissions to the NICU: (*F*_(1, 29)_ = 25.37, *p* < 0.001, $\eta_{\text{p}}^{2}$ = 0.47, 95%CI[−0.66, −0.28]) from pre-test (*M* = 0.0, *SD* = 0.0) to post-test (*M* = 0.47, *SD* = 0.51). While none of the HCPs recommended admitting the baby to the NICU as an option on the pre-test, the post-test identified a significant improvement in NICU admissions. We believe, that HCPs did not consider NICU admissions as an action during the pre-test-scenario and rather focused on the stabilization of the infant. However, within the RETAIN simulator, all scenarios end with either “*admission to the NICU”* or “*give baby to mother,”* which might have resulted in the improved score.

## Discussion

We report the use of a specifically-developed educational neonatal resuscitation board game to facilitate learning and its effect on short-term knowledge retention in HCPs. Overall, we observed a 12% increase in performance between the pre- and post-test (49–61%, respectively). Temperature management showed the most knowledge gain between the pre- and post-test (14–46%, respectively). Placement of a hat (10–43%), plastic wrap (27–67%), and temperature probe (7–30%) improved between the pre- and post-test. Also, attaching the SpO_2_ (57–77%) and attaching the ECG (47–73%) improved from the pre-test to the post-test. Finally, starting Continuous Positive Airway Pressure (80–97%) and admitting to NICU (0–47%) also improved from the pre-test to the post-test. The results of the study can be summarized as follows: (i) the board game significantly improved participants' performance on a specific neonatal resuscitation scenario between pre- and post-test; (ii) temperature management and cardiorespiratory monitoring were applied significantly more often; (iii) although breathing assessment was poorer on the post-test, the timing of starting respiratory support was significantly improved; and (iv) this is the first time, to our knowledge, that a simulator has used evidence-based neonatal resuscitation scenarios.

Hypothermia at admission occurs between 32and 85% of cases (15, 16), and for each 1°C decrease in admission temperature, mortality increases by 28% (OR: 1.28; CI: 1.16–1.41) and late-onset sepsis by 11% (OR: 1.11; CI: 1.02–1.20) in low birth weight infants (17). Studies examining heat-loss prevention reported that plastic wraps or bags, plastic caps, heated mattresses, and skin-to-skin contact keep infants warmer than routine preventative action (18–21). While these interventions can prevent heat-loss, studies auditing delivery room practices reported that they are only applied in 58–79% of cases within the timeline of the current resuscitation guidelines (3, 4). In the board game, we observed an overall improvement of adequate temperature management from 14 to 46% between the pre- and post-test. Similarly, placement of a hat increased from 10 to 43%, using a plastic wrap from 27 to 67%, and attaching a temperature probe from 7 to 30% between the pre- and post-test. This suggests that the RETAIN board game has the potential to improve HCPs temperature management. Similar improvements were seen with cardiovascular monitoring including pulse oximetry and electrocardiography.

Our results are similar to those collected with previous training board games including *Neonatology, Neonopoly*, or *The Neonatal Emergency Trivia Game* (22–24). Furthermore, a randomized, controlled trial assessing the effectiveness of the *Neonatology* game revealed that the participants that played the game (*n* = 31) averaged 4.14 points better on true and false exams pertaining to the content (95% CI−0.88–9.17; *p* = 0.09) than the students that did not play the game (*n* = 37) (24). Midwives using the board game *Neonopoly* reported that it helped to make the material more interesting, and 94% of the participants enjoyed playing that game (23). Furthermore, Gordon et al. used a five-point Likert Scale to assess applicability for the neonatal work setting, which was rated between 4.2 and 4.8 (22). Board or video games can be effective tools for teaching information to various learners in different situations (10, 14, 25, 26). However, games are not suitable for presenting all types of materials. Games do not lend themselves to the delivery of large amounts of information over short periods of time (25, 26). However, games have been found to be a very effective method of reviewing material and reinforcing facts and utilizing the knowledge gained through individual experiences and enhancing motivation by providing an avenue for validating personal knowledge and sharing experiences with peers (25, 26). Participants are able to address learning needs that are relevant to their individual practice as they interact in small-group discussions to solve simulated problems (25, 26). Games also allow HCPs to practice with rare cases, including high-acuity low-observance (HALO) situations. Therefore, gaming might be an effective tool that can be used to present educational material to adult learners in a manner that addresses their learning needs.

A major advantage of the RETAIN board game simulator is the use of evidence-based neonatal resuscitation scenarios, which are scenarios translated from the delivery room. Everything within the case, including maternal and pregnancy history, along with the course of the infant over the first 10 min in the delivery room, the interventions that had been done, and changes in heart rate and oxygen saturation originate from real-life deliveries. This is an important advancement in this context, as scenarios are generally made up by instructors or educators and changed as the simulation progresses. Using evidence-based neonatal resuscitation scenarios allows the facilitator to focus on tasks related to learning and support. Additionally, the tactile nature of the board game interaction may play a role in the performance improvement demonstrated by this intervention (e.g., the game facilitated the use of props for a hat, plastic wrap, temperature probe, etc.).

There are several limitations of board games in general, which should be noted. In particular, a board game would need (i) a dedicated space to play the game, which could be even a lunch room; (ii) some set-up time, which is generally short (~5 min); (iii) a facilitator, who uses the booklet to guide the player through the scenario; and (iv) several players to play the game. Despite HCPs spending a relatively short time playing the *RETAIN* neonatal resuscitation board game, their performance was improved. However, we identified some aspects that could be further improved. Namely, participants' assessment of breathing skill deteriorated from pre- to post-test. There are many other aspects, which could be tested using the *RETAIN* board game including *cardiovascular* (e.g., chest compressions), v*enous access* (e.g., umbilical catheter or intraosseous access), *medication* (e.g., administration of epinephrine), or *post resuscitation* care (e.g., admission or transfer to mother). While these are all very important aspects of neonatal resuscitation, the current study did not examine them, which is a limitation of the study. Future studies will examine these aspects of the game more closely and potentially adjust the design of the game. Finally, another study will ask participants to play both the current board game version and the online version of the simulation to collect learning analytics and to compare performance in both media to test skill transfer from one medium to another.

## Limitation

This study included a sample of convenience of 30 HCPs, which is limitation of the study. The small sample precluded subgroup analyses according to HCP professions, which will be addressed in a future study by collecting additional data. The post-test was assessed immediately after playing the game, and not at a later time point (e.g., 3-months after playing the game). In future study, a different post-test will be administered after a delay. Furthermore, we only assessed cardiorespiratory assessment as attachment of pulse oximetry and electrocardiography leads. Unfortunately, we did not assess the frequency of cardiorespiratory assessment throughout the game play, which is another limitation of the study that will be addressed in a follow-up study.

## Conclusions

The RETAIN board game significantly improved participants' performance and knowledge on a specific neonatal resuscitation scenario between pre- and post-test. The improvement in performance and knowledge supports the use of board game simulations for clinical training. Further studies should include a larger number of participants from a variety of healthcare professions and should be conducted in different healthcare systems and different cultures to assess the acceptance of the game.

## Author Contributions

GS, MC, MB, and PvH: conception and design; GS, MC, MB, CF, SP, TJ, and PvH: collection and assembly of data; GS, MC, MB, CF, SP, TJ, and PvH: Analysis and interpretation of the data; GS, MC, MB, CF, SP, TJ, and PvH: drafting of the article; GS, MC, MB, CF, SP, TJ, and PvH: Critical revision of the article for important intellectual content; GS, MC, MB, CF, SP, TJ, and PvH: final approval of the article.

### Conflict of Interest Statement

MB, PvH, and GS have registered the game under Canadian copyright [Tech ID 2017083; https://www.playretain.com], and MB and GS are owners of RETAIN Labs Medical Inc., which is distributing the game. The remaining authors declare that the research was conducted in the absence of any commercial or financial relationships that could be construed as a potential conflict of interest.

## Abbreviations

NRP: Neonatal resuscitation program
SBME: Simulation-based medical education
HCP: Healthcare provider.
