# Supplementary material for: RETAIN: A Board Game That Improves Neonatal Resuscitation Knowledge Retention
Source: Front Pediatr. 2019 Jan 31;7:13. doi: 10.3389/fped.2019.00013 (PMC6365420; doi:10.3389/fped.2019.00013)
Supplement: Supplementary file 1 [file Data_Sheet_1.docx]

**Appendix 1: The scenario and answers for the pre-test and post-test**

Scenario: Your task is to identify the steps needed to react in the following situation: a newborn baby with fetal distress (fetal heart rate abnormalities) is brought to you. What are the steps you would need to take in order of highest to lowest priority?

26-year-old woman Gravida 1 Para 0 with no prenatal care currently at 24 weeks pregnant. The Mother received 2 doses of Betamethasone and Magnesium sulfate prior caesarean section due to fetal heart abnormalities. The baby is delivered and receives 60sec of delayed cord clamping. The baby is transferred to the Resuscitation table.

*What are the next steps?*

1. Hat
2. Wrap
3. Stimulate
4. Visually assess
5. Assess breathing
6. Assess heart rate

The clinical team performs initial assessment: Heart rate 70 beats/min, blue skin color, and apnea

*What are the next steps?*

1. Attach pulse oximetry sensors
2. Attach Electrocardiography leads
3. Attach temp probe
4. Suction
5. Reposition head

After reassessment: Heart rate 50 beats/min, blue skin color, and apnea

*What are the next steps?*

1. Positive Pressure Ventilation for 60 sec

After reassessment: Heart rate >100/min, apnea

*What are the next steps?*

1. MR. SOPA
2. Positive Pressure Ventilation for 60 sec

After reassessment: Heart rate >120 beats/min, spontaneous breathing with increased work of breathing and grunting

*What are the next steps?*

1. Start Continuous Positive Airway Pressure
2. Admit to Neonatal Intensive Care Unit

No further actions are required thereafter

Abbreviation: MR. SOPA - [M (mask adjustment), R (reposition airway), S (suction mouth and nose), O (open mouth), P (pressure increase), A (alternate airway]
